# *Zanthoxylum bungeanum* Essential Oil Nanoemulsion: Preparation, Characterization, and Insecticidal Activity Against *Tribolium castaneum*

**DOI:** 10.1002/cbdv.71749

**Published:** 2026-09-27

**Authors:** Xu‐Dong Wang, Zhi‐Qin Liu, Xue Fan, Jia‐Li Bai, Jun‐Yu Liang, Ji Zhang

**Affiliations:** ^1^ College of Life Science Northwest Normal University Lanzhou Gansu P.R. China; ^2^ New Rural Development Research Institute Northwest Normal University Lanzhou Gansu P.R. China

**Keywords:** contact toxicity, fumigant toxicity, physicochemical stability, repellency, stored‐product pest

## Abstract

*Zanthoxylum bungeanum* essential oil (ZBEO) is a promising botanical insecticide, but its volatility, poor stability, and low water solubility limit practical application. This study developed a ZBEO nanoemulsion (ZBEO‐NE) and evaluated its physicochemical properties, insecticidal activity, repellency, stability‐related bioactivity retention, and cytotoxicity. GC–MS identified linalool (53.78%) as the major component. The resulting ZBEO‐NE had a mean droplet size of 128.90 nm, a polydispersity index of 0.20, and a zeta potential of −41.90 mV. TEM revealed uniformly distributed spherical droplets, and rheological analysis showed shear‐thinning behavior. The formulation remained physically stable during 30 days of storage and after heating and centrifugation. Against *Tribolium castaneum* adults and larvae, ZBEO‐NE showed lower contact toxicity than free ZBEO, whereas its fumigant activity increased with exposure time. LC_50_ values decreased from 20.62 to 12.68 mg/L air in adults and from 14.05 to 7.06 mg/L air in larvae between 24 and 72 h. ZBEO‐NE also showed more persistent repellency and largely retained biological activity after stability treatments, although heating at 100°C reduced adult fumigant mortality. Cytotoxicity was concentration dependent, with good cytocompatibility at relatively low concentrations. These findings support ZBEO‐NE as a potential formulation for stored‐product pest management, with promise for practical stored‐product pest control applications.

## Graphical abstract

*Zanthoxylum bungeanum* essential oil is formulated as a stable nanoemulsion with nanoscale droplets, strong colloidal stability, and shear‐thinning behavior. The formulation retains insecticidal and repellent activity after storage and stress treatments, shows time‐dependent fumigant efficacy against *Tribolium castaneum* adults and larvae, and exhibits good cytocompatibility at relatively low concentrations.

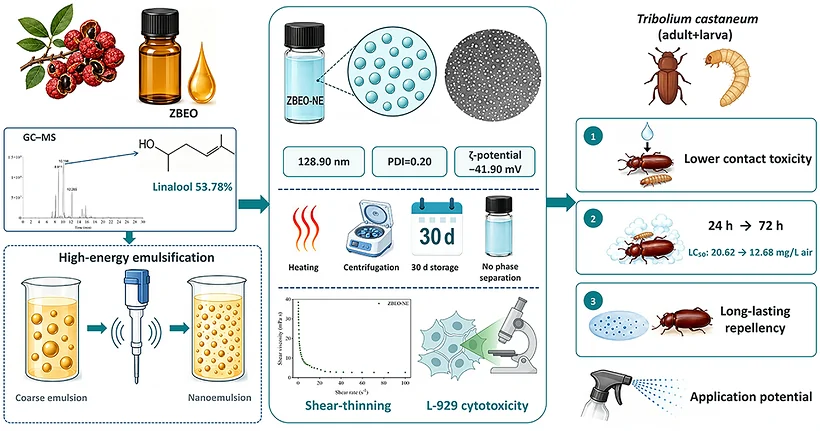

## 1 Introduction

Stored‐product insect pests represent a major global threat to food security by contaminating grains, processed foods, and other commodities throughout international distribution systems, resulting in substantial economic losses [1]. Among these pests, *Tribolium castaneum* (Herbst, 1797) (Coleoptera: Tenebrionidae) is recognized as one of the most significant species affecting stored products worldwide, causing an estimated annual loss of 10%–15% in stored commodities [2]. This species causes considerable agricultural and economic damage through direct infestation of grains and their by‐products, leading to weight loss, quality deterioration, and reduced marketability [3]. In addition, infestations produce hazardous compounds that persist on contaminated materials, posing long‐term risks to stored‐product safety [4]. The presence of *T. castaneum* may also facilitate fungal proliferation and subsequent mycotoxin contamination, further compromising food safety [5]. Consequently, this species is regarded as a major pest that requires continuous monitoring and effective control measures [6].

The management of *T. castaneum* and other stored‐product pests relies predominantly on synthetic insecticides, particularly phosphine [7]. However, the frequent application of high doses to counteract insect resistance substantially increases treatment costs, environmental contamination, and risks to human health [8]. Therefore, the development of sustainable and environmentally friendly alternatives is urgently needed to strengthen integrated pest management strategies for stored‐product insects [9]. Botanical insecticides have gained increasing attention as biodegradable alternatives that leave minimal toxic residues [10]. Among these, plant essential oils (EOs) are the most extensively investigated class of botanical pesticides and are characterized by high specificity, rapid environmental degradation, and relatively low toxicity to mammals and nontarget organisms [11].

*Zanthoxylum bungeanum*, a widely distributed Asian plant commonly used in traditional Chinese medicine, is valued for its high EO content [12]. *Z. bungeanum* EO (ZBEO) exhibits notable antibacterial, anti‐inflammatory, and insecticidal activities, highlighting its potential for applications in the pharmaceutical and agricultural sectors [13, 14]. Although plant‐derived EOs represent promising candidates for stored‐product pest management due to their complex chemical composition and multitarget modes of action, their practical application remains limited by several inherent limitations [15]. In particular, high volatility, poor physicochemical stability, and low water solubility contribute to reduced bioavailability and short residual activity [16]. To address these limitations, encapsulation within nanoemulsion (NE) systems has been proposed as an effective formulation strategy [15].

Nanoemulsions (NEs) are colloidal dispersion systems composed of two immiscible liquids, in which one phase is dispersed as nanoscale droplets (20–200 nm) within the other, forming a kinetically stable and homogeneous system [17]. Compared with unformulated EOs, EO‐based NEs exhibit improved water solubility and enhanced bioactivity [18]. For example, cinnamon oil NEs demonstrated higher efficacy against *Sitophilus oryzae* than the free oil [9]. Similarly, NEs of *Eucalyptus globulus* EO exhibited enhanced insecticidal activity against *T. castaneum* [19]. Although the insecticidal activity of ZBEO against *T. castaneum* adults has been previously reported, studies investigating its NE formulation remain limited [20]. Therefore, we aimed to prepare ZBEO‐NE, characterize its physicochemical properties, and compare the contact, fumigant, and repellent activities of ZBEO and ZBEO‐NE against *T. castaneum*. This work provides a possible method for improving EO‐based insecticides.

## 2 Materials and Methods

### 2.1 Plant Material

The *Z. bungeanum* material used in this study was purchased on July 15, 2025, from Tianxiang Technology Co., Ltd. (Cheng County, Longnan City, Gansu Province, China). The plant material was authenticated as *Z. bungeanum* Maxim. by Professor Junyu Liang (College of Life Sciences, Northwest Normal University, Lanzhou, China). A voucher specimen was deposited in the Herbarium of the College of Life Sciences, Northwest Normal University, Lanzhou, China, under the voucher number ZB‐20250715‐010. The samples used for essential oil extraction were selected based on uniform coloration and the absence of physical damage or insect infestation.

### 2.2 EO Extraction

EO was obtained from 500 g of dried, milled *Z. bungeanum* pericarps by hydrodistillation using a Clevenger‐type apparatus with 5 L of distilled water, following a previously reported method with minor modifications [21]. The mixture was heated to gentle boiling and maintained under continuous reflux for 2 h. The yield was calculated based on the dry weight of the plant material. After collection, the oil was dehydrated over anhydrous Na_2_SO_4_, weighed, and stored in sealed amber vials at 4°C for up to 2 weeks before use.

$$\mathrm{Yield}\;\mathrm{equation}\;(\%)=\frac{\mathrm{EOs}\;\mathrm{weight}\;(g)\;}{\mathrm{Sample}\;\mathrm{weight}\;(g)}\;\times \;100$$

### 2.3 Gas Chromatography‐Mass Spectrometry (GC‐MS) Analysis

The chemical composition of ZBEO was determined using the Agilent 7890A GC system coupled with an Agilent 5975C MS detector, equipped with an HP‐5MS capillary column (30 m × 250 µm × 0.25 µm; Agilent Technologies). The oven temperature was maintained at 60°C for 2 min, increased to 180°C at 5°C/min and held for 1 min, then increased to 280°C at 10°C/min and held for 10 min. A 1 µL aliquot of 1% sample solution (in hexane) was then injected at 270°C in split mode (1:100). High‐purity helium (≥99.999%) was used as the carrier gas at a constant flow rate of 1.0 mL/min. The retention indices (RI) were experimentally determined under the same chromatographic conditions as those used for sample analysis. A series of C_8_–C_24_ *n*‐alkane standards was injected under identical GC‐MS conditions, and the retention times were recorded. The RI values for each compound were calculated using the Van den Dool and Kratz equation for temperature‐programmed gas chromatography.

$$\mathrm{RI}\;\mathrm{equation}=100\;\times \;C+100\;\times \;\frac{T_{Z}\;-\;T_{C}}{T_{C+1}\;-\;T_{C}}$$

where C denotes the number of carbon atoms in *n*‐alkane; T_Z_ represents the retention time of the test compound; T_C_ denotes the retention time of the preceding *n*‐alkane; and T_C+1_ denotes the retention time of the subsequent *n*‐alkane. Individual constituents were identified using a dual‐criteria approach. First, the experimentally determined RI values were compared with those reported in the published literature, with a deviation threshold of ± 10 RI units accepted for identification. Second, the mass spectra of each compound were matched against the NIST23 mass spectral library, with a minimum match factor of 90% required for confirmation. Only constituents satisfying both RI and MS matching criteria were considered identified.

### 2.4 NE Preparation

The preparation of ZBEO‐NE followed a high‐energy emulsification method adapted from [22] with some modifications. The oil phase was composed of 10% (w/w) ZBEO, whereas the aqueous phase contained a mixed surfactant system (3% w/w; Tween‐80 1.39 g, Span 80 0.14 g) and 1.5% (w/w) anhydrous ethanol dissolved in deionized water, with a total batch mass of 50 g. The oil phase was added dropwise to the aqueous phase under continuous stirring. Primary emulsification was performed by magnetic stirring at 350 rpm for 2 h, followed by high‐speed homogenization at 10 000 rpm for 3 min. The resulting coarse emulsion was sonicated using a probe ultrasonicator (JY92‐IIN, Ningbo Scientz Biotechnology Co., Ltd., Ningbo, China) at 455 W for 5 min in pulse mode (2 s on/2 s off), while the sample vessel was immersed in an ice‐water bath to minimize heat accumulation.

### 2.5 Characterization of NEs

#### 2.5.1 Particle Size, Polydispersity Index (PDI), and Zeta Potential

The colloidal characteristics of the NEs, including particle size, PDI, and zeta potential, were measured in triplicate by using a Litesizer DLS 500 instrument (Anton Paar) maintained at 25°C. The analytical procedure was adapted from a previous study [23] with specific modifications. Before measurement, each sample was diluted 100‐fold with deionized water.

#### 2.5.2 Transmission Electron Microscopy (TEM)

The morphology of NE droplets was examined by TEM (Hitachi 7800) at 120 kV by following a modified method from a previous study [24]. Briefly, a diluted sample was deposited onto a carbon‐coated copper grid, blotted with filter paper, and negatively stained with 2% (w/v) phosphotungstic acid for 30 s. After rinsing with deionized water and air‐drying at room temperature, micrographs were acquired using an integrated digital camera.

#### 2.5.3 Rheological Analysis

The rheological behavior of the ZBEO‐NE was characterized by using an MCR 302e rheometer (Anton Paar) at 25°C, as per a methodology reported elsewhere [25]. Steady‐state flow measurements were performed across a shear rate spectrum of 0.01–100 s^−1^ to establish the viscosity profile of the formulation.

### 2.6 Stability Studies

#### 2.6.1 Storage Stability

To evaluate the colloidal stability of the ZBEO‐NE, samples were maintained in triplicate in sealed 2‐mL centrifuge tubes at 4°C and 25°C in accordance with an adapted protocol from a previous study [26]. The assessment involved tracking alterations in particle size, PDI, and zeta potential at 0, 15, and 30‐day intervals, complemented by visual examination for phase separation.

#### 2.6.2 Thermal Stability

ZBEO‐NE samples in triplicate underwent thermal treatment at 4, 25, 50, 75, and 100°C for 30 min as per an adapted methodology [27]. After achieving an equilibrium at ambient temperature, post‐treatment analysis included the measurements of particle size and zeta potential for the evaluation of thermal stability.

#### 2.6.3 Centrifugal Stability

ZBEO‐NE samples in triplicate were centrifuged at progressively higher speeds of 2 000, 4 000, 6 000, 8 000, and 10 000 rpm, with each speed maintained for 10 min, as per the method reported elsewhere [28]. Comparative analysis of the particle size and zeta potential was conducted before and after each centrifugation cycle to evaluate the centrifugal stability.

### 2.7 Insect Rearing

*Tribolium castaneum* adults and larvae were obtained from laboratory cultures maintained in continuous darkness at 29 ± 1°C and 70 ± 10% relative humidity. The insects were reared in glass vials (2 cm in diameter and 8 cm in height) containing a diet of wheat flour and brewer's yeast (10:1, w/w) with a moisture content of 13 ± 1%. Healthy and active adults aged 1–2 weeks post‐eclosion and 6‐day‐old larvae were selected for the bioassays. The sexes of the adults were not separated.

### 2.8 Bioassay Methods

#### 2.8.1 Contact Toxicity

The contact toxicities of ZBEO and ZBEO‐NE against *T. castaneum* adults and larvae were evaluated as per the method of [29] with modifications. For the EO, serial dilutions were prepared in acetone, whereas serial dilutions of the NEs were prepared in deionized water alone. Five test concentrations [8%, 4%, 2%, 1%, and 0.5% (w/v)] were prepared for each formulation at equivalent EO concentrations, with five replicates per concentration. A 0.5 µL aliquot of each dilution was then topically applied to the dorsal thorax of insects that were briefly anesthetized by cold exposure. The treated insects were then placed in glass vials (2 cm × 8 cm) with 10 insects/vial. Pyrethrin was used as the positive control. Acetone served as the negative control for the EO treatment, and a blank NE (prepared with the same surfactant concentration but without EO) served as the negative control for the NE treatment. Based on the applied volume of 0.5 µL per insect, the test concentrations corresponded to doses of 40, 20, 10, 5, and 2.5 µg/insect, respectively. Mortality was recorded at 24, 48, and 72 h post‐treatment.

#### 2.8.2 Fumigant Toxicity

The fumigant toxicity of ZBEO and ZBEO‐NE against *T. castaneum* adults and larvae was evaluated following the method of [29] with some modifications. For the EO, serial dilutions were prepared in acetone, while for the NEs, serial dilutions were prepared in deionized water alone. Five initial test concentrations [8%, 4%, 2%, 1%, and 0.5% (w/v)] were prepared for each formulation at equivalent EO concentrations. A 10 µL aliquot of each dilution was applied to a filter paper (2.0 cm diameter), which was then attached to the inner surface of a screw cap. Based on the vial volume (approximately 0.025 L), the amounts of EO delivered corresponded to fumigation concentrations of 32, 16, 8, 4, and 2 mg/L air, respectively. The cap was tightly sealed onto a glass vial (2 cm × 8 cm) containing 10 insects. Phosphine and acetone served as positive and negative controls, respectively, for the EO treatment. For the NE treatment, a blank NE was used as the negative control. Insects showing no movement of legs or antennae were considered dead. Mortality was recorded at 24, 48, and 72 h post‐treatment.

#### 2.8.3 Repellency Activity

The repellent activities of ZBEO and ZBEO‐NE against *T. castaneum* adults were evaluated by using a filter paper‐based choice bioassay according to the methods of [30] with some modifications. For the EO, serial dilutions were prepared in acetone, while for the NEs, serial dilutions were prepared in deionized water alone. The test concentrations were selected based on preliminary range‐finding bioassays to cover a broad range of repellent responses. Five concentrations of 78.63, 15.73, 3.15, 0.63, and 0.13 nL/cm^2^ were prepared by fivefold serial dilution and expressed as equivalent EO doses per unit area. Each concentration was tested with five independent replicates. A filter paper (9 cm in diameter) was cut into two equal semicircles. One semicircle was uniformly treated with 500 µL of the test solution, while the other semicircle received the same volume of the corresponding control solvent (acetone for the EO tests, and a blank NE for the NE tests). The two semicircles were reassembled and placed at the bottom of a Petri dish (9‐cm diameter). A group of 20 insects was released at the center of the filter paper, and the dish was covered to allow them to move freely. The number of insects present in the treated and control semicircles was recorded after 2, 4, 6, 12, 24, 48, and 72 h of exposure. The repellent performance was evaluated based on the calculated preference rate (%).

$$\mathrm{Preference}\;\mathrm{equation}\;(\%)=\frac{C}{T+C}\;\times \;100$$

where C represents the number of adults selecting the control side, and T represents the number of adults selecting the ZBEO/ZBEO‐NE‐treated side.

### 2.9 Biological Activity After Stability Treatments

To evaluate the retention of biological activity after stability treatments, initial ZBEO‐NE and samples subjected to storage, heating, or centrifugation were tested. The storage‐treated samples were maintained at 4°C or 25°C for 30 days. The heat‐treated samples were exposed to 100°C for 30 min and subsequently cooled to room temperature, whereas the centrifugation‐treated samples were centrifuged at 10 000 rpm for 10 min. Contact and fumigant toxicities were evaluated against both adults and larvae of *T. castaneum* at the highest tested doses of 40 µg/insect and 32 mg/L air, respectively. Repellency was evaluated against adults at 78.63 nL/cm^2^. All bioassays were performed following the procedures described above, and mortality or repellency was recorded at 72 h. Each treatment was independently replicated five times.

### 2.10 Cytotoxicity Assay

L‐929 fibroblasts were cultured in Dulbecco's modified Eagle's medium supplemented with 10% fetal bovine serum and 1% penicillin–streptomycin at 37°C in a humidified atmosphere containing 5% CO_2_. Cells in the logarithmic growth phase were seeded into 96‐well plates and allowed to attach for 48 h. The culture medium was then replaced with fresh medium containing ZBEO‐NE at concentrations of 1.0, 0.5, 0.25, 0.125, and 0.0625 mg/mL. Cells cultured without ZBEO‐NE served as the control. After 24 h of exposure, cell viability was determined using the CCK‐8 assay according to a previously reported method with minor modifications [31]. Absorbance was measured at 450 nm using a microplate reader.

### 2.11 Statistical Analysis

Mortality data from the contact and fumigant toxicity assays were analyzed by probit analysis using IBM SPSS Statistics 27.0 to estimate LD_50_, LD_90_, LC_50_, and LC_90_ values with 95% confidence intervals. Insect distributions in the repellency assay were analyzed using chi‐square tests against a theoretical 50:50 distribution. Other data were analyzed by one‐way ANOVA followed by Duncan's multiple‐range test. Differences were considered significant at *p* < 0.05. Figures were prepared using GraphPad Prism 9.5.

## 3 Results

### 3.1 Yield of EOs

As shown in Table 1, hydrodistillation of dried *Z. bungeanum* pericarps produced an EO yield of 4.88% under the present extraction conditions.

**TABLE 1:** Extraction yields of EOs from *Z. bungeanum*.

Samples	Sample weight (g)	EO's weight (g)	Extraction rate (%)
*Z. bungeanum*	500	24.40	4.88

### 3.2 Chemical Composition of EOs

**FIGURE 1:**
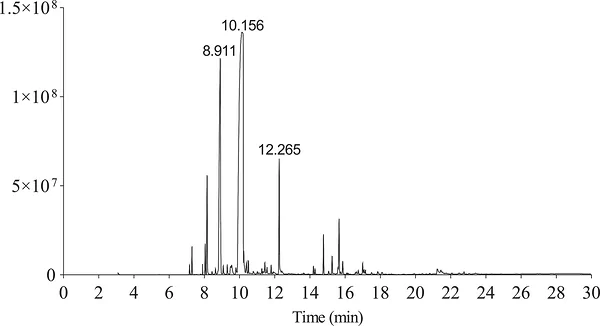
Representative total ion current (TIC) chromatogram of ZBEO obtained by GC‐MS analysis.

EOs are complex phytochemical mixtures whose bioactivities depend largely on the relative abundance of their individual components. To characterize the chemical composition of ZBEO, the oil was analyzed by GC‐MS. A total of 19 volatile compounds were identified, collectively accounting for 87.96% of the total detected composition (Table 2 and Figure 1). The volatile profile was dominated by linalool (53.78%), followed by limonene (17.16%) and linalyl acetate (5.90%). These three compounds collectively accounted for 76.84% of the total detected composition.

**TABLE 2:** Chemical composition of ZBEO.

No.	RT	RI^a^	RI^b^	Match scores	Compounds	Formula	Relative content (%)
1	7.165	932	931	98.11	*α*‐Thujene	C_10_H_16_	0.37
2	7.304	940	939	98.85	*α*‐Pinene	C_10_H_16_	0.86
3	8.162	994	981	98.17	*β*‐Pinene	C_10_H_16_	3.86
4	8.640	1023	1018	97.52	4‐Carene	C_10_H_16_	0.29
5	8.911	1040	1044	98.19	Limonene	C_10_H_16_	17.16
6	9.081	1051	1050	98.56	*β*‐Ocimene	C_10_H_16_	0.38
7	9.310	1065	1061	98.83	*γ*‐Terpinene	C_10_H_16_	0.31
8	9.491	1076	1070	98.89	Sabinene hydrate	C_10_H_18_O	0.38
9	9.552	1080	1076	93.42	2‐Furanmethanol	C_10_H_18_O_2_	0.68
10	10.156	1118	1117	98.51	Linalool	C_10_H_18_O	53.78
11	10.253	1125	1116	97.91	4‐Thujanol	C_10_H_18_O	0.78
12	10.404	1135	1126	99.34	*β*‐Thujone	C_10_H_16_O	0.54
13	10.506	1141	1135	96.54	Dihydrolinalool	C_10_H_20_O	0.46
14	11.449	1202	1198	97.27	*α*‐Terpineol	C_10_H_18_O	0.44
15	12.265	1259	1259	98.51	Linalyl acetate	C_12_H_20_O_2_	5.90
16	15.262	1475	1465	99.27	Humulene	C_15_H_24_	0.52
17	15.872	1519	1511	98.04	Bicyclogermacrene	C_15_H_24_	0.50
18	16.748	1581	1580	94.22	Cyclohexadecane	C_16_H_32_	0.36
19	17.074	1605	1605	96.06	Spathulenol	C_15_H_24_O	0.39

RI^a^ values were experimentally determined using the C_8_–C_24_ *n*‐alkane series under identical analytical conditions.

RI^b^ reported in previous studies.

Compound identification required both RI matching (± 10 units) and MS spectral agreement (≥ 90% match factor) with published literature and NIST23 database.

### 3.3 Characterization of NEs

#### 3.3.1 Particle Size, PDI, and Zeta Potential

The freshly prepared ZBEO‐NE appeared as a homogeneous, milky‐white liquid retaining the characteristic aroma of the parent EO. The mean droplet size, PDI, and zeta potential were 128.90 nm, 0.20, and −41.90 mV, respectively, as summarized in Table 3.

**TABLE 3:** Physicochemical characteristics of ZBEO‐NE.

Parameter	ZBEO‐NE
Mean droplet size (nm)	128.90 ± 3.44
PDI	0.20 ± 0.01
Zeta potential (mV)	−41.90 ± 0.61

Values represent the mean ± standard deviation of three replicates (*n* = 3).

#### 3.3.2 TEM

**FIGURE 2:**
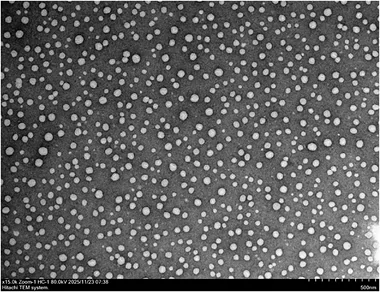
TEM image showing spherical nanodroplets of ZBEO‐NEs.

TEM was used to visualize the nanoscale morphology of the ZBEO‐NE formulation. As shown in Figure 2, the NE droplets exhibited spherical morphology with a relatively uniform size distribution. Image analysis indicated droplet diameters of approximately 100 nm. No evidence of droplet aggregation or coalescence was observed in the micrographs.

#### 3.3.3 Rheological Analysis

**FIGURE 3:**
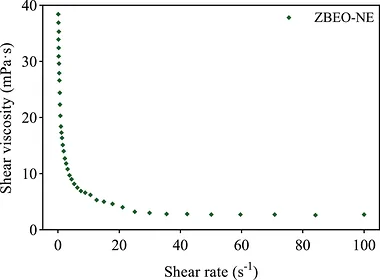
Rheological characteristics of ZBEO‐NE.

The rheological properties of ZBEO‐NE are presented in Figure 3. The formulation exhibited non‐Newtonian flow behavior characterized by shear‐thinning, where the apparent viscosity decreased with increasing shear rate. A critical shear rate of approximately 2 s^−1^ was observed, below which viscosity declined sharply before approaching a plateau at higher shear rates.

### 3.4 Stability Studies

#### 3.4.1 Storage Stability

**FIGURE 4:**
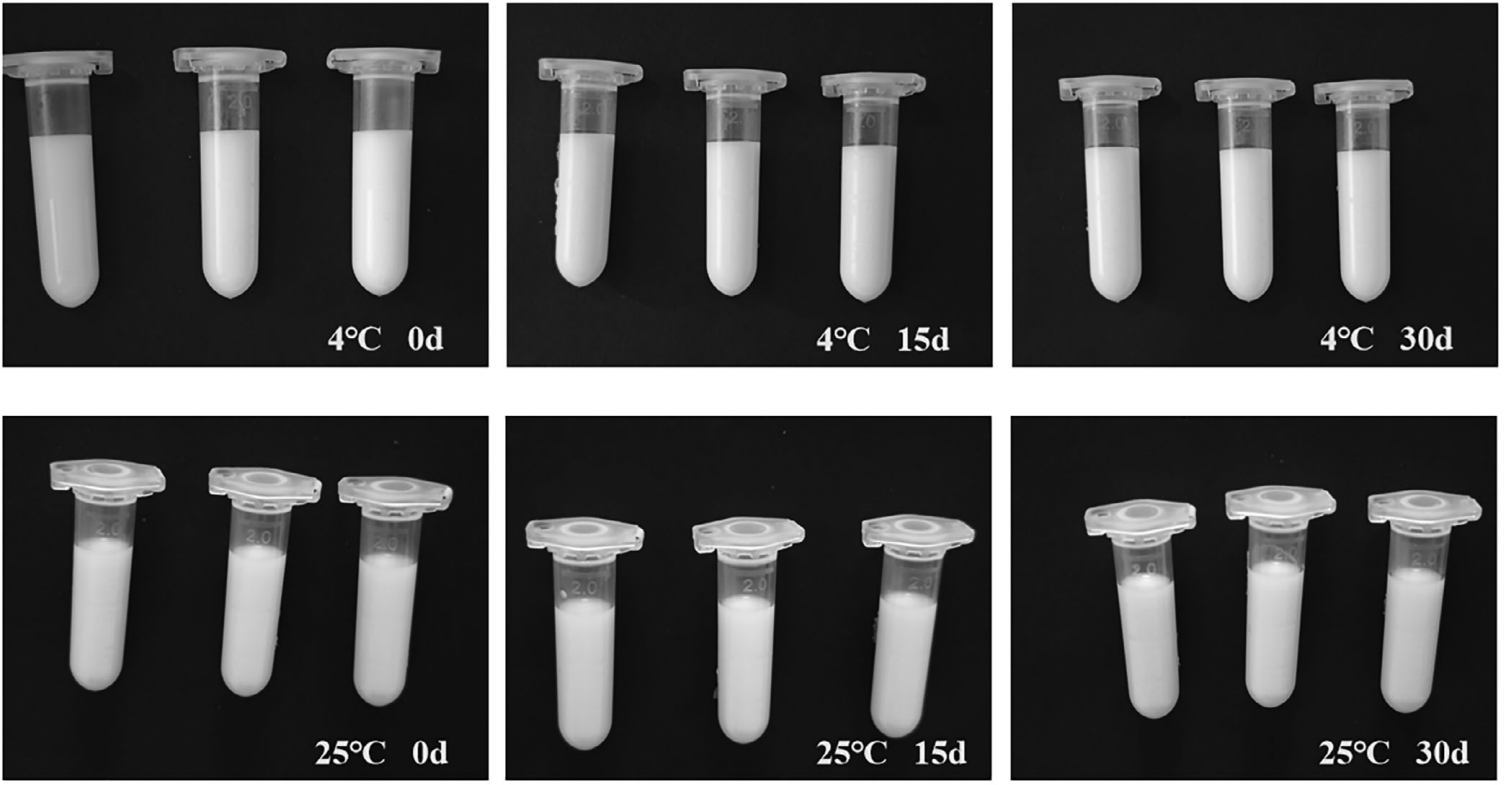
Photographs of ZBEO‐NE during storage at 0, 15, and 30 days.

The storage stability of ZBEO‐NE was evaluated over 30 days at 4 and 25°C. Throughout the storage period, the formulation maintained a homogeneous, milky‐white appearance without visible phase separation at either temperature (Figure 4). Particle size, PDI, and zeta potential were measured at 15‐day intervals, and the complete dataset is presented in Table 4. As summarized in Table 4, at 4°C the mean particle size changed from 128.90 nm (0 days) to 116.40 nm (30 days), while PDI ranged from 0.20 to 0.23 and the zeta potential varied from −41.90 to −38.92 mV. At 25°C, particle size decreased from 128.90 to 115.80 nm, with PDI ranging from 0.19 to 0.25 and zeta potential changing from −41.90 to −35.35 mV.

**TABLE 4:** Stability of ZBEO‐NE at 4°C and 25°C for different storage times.

T (°C)	Days (d)	Size (nm)	PDI	Zeta potential (mV)
4	0	128.90 ± 3.44^a^	0.20 ± 0.01^a^	−41.90 ± 0.61^b^
	15	120.30 ± 1.92^b^	0.24 ± 0.04^a^	−37.37 ± 0.91^a^
	30	116.40 ± 2.77^b^	0.23 ± 0.02^a^	−38.92 ± 1.02^a^
25	0	128.90 ± 3.44^a^	0.20 ± 0.01^ab^	−41.90 ± 0.61^b^
	15	122.70 ± 4.21^ab^	0.19 ± 0.04^c^	−40.21 ± 2.47^b^
	30	115.80 ± 3.03^b^	0.25 ± 0.01^a^	−35.35 ± 0.26^a^

Values represent the mean ±  standard deviation of three replicates. Different lowercase letters within the same column at the same temperature indicate significant differences among storage durations (0, 15, and 30 d) (*n* = 3, *p* < 0.05).

#### 3.4.2 Thermal Stability and Centrifugal Stability

**FIGURE 5:**
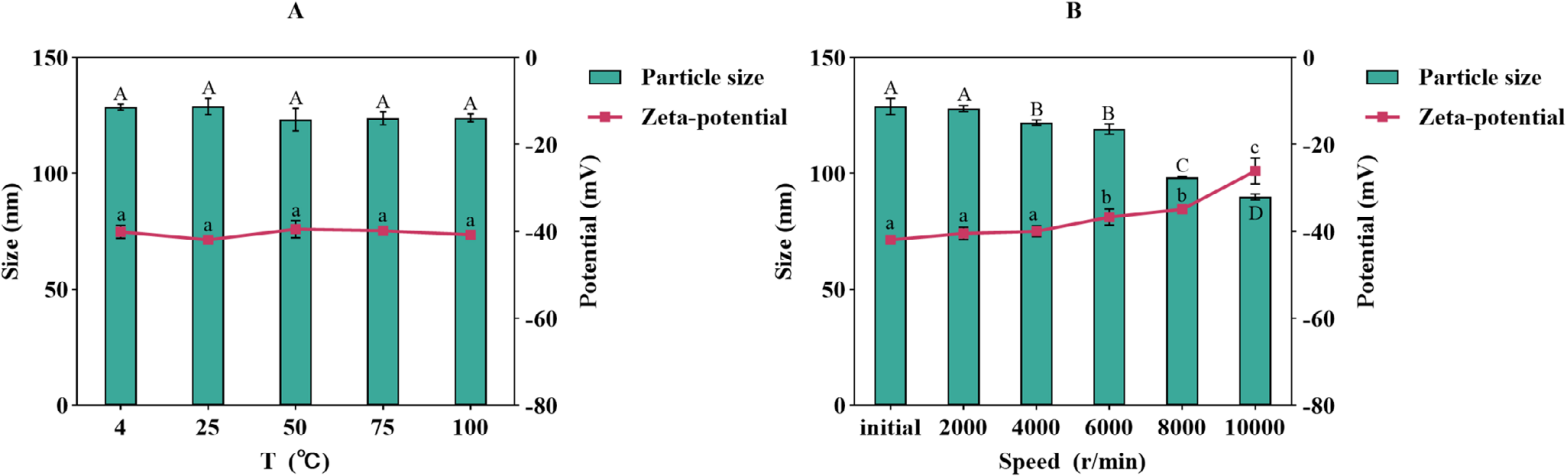
Thermal (A) and centrifugal (B) stability of ZBEO‐NE. Values are presented as means ± SD (*n* = 3, *p* < 0.05).

Thermal stability of ZBEO‐NE was evaluated by measuring changes in particle size and zeta potential after exposure to temperatures of 4°C, 25°C, 50°C, 75°C, and 100°C for 30 min. As shown in Figure 5, both parameters remained largely unchanged across the tested temperature range, indicating strong thermal stability of the NE. Centrifugal stability testing revealed a threshold effect for the ZBEO‐NE formulation. At rotational speeds below 4000 rpm, the NE maintained its colloidal characteristics, with particle size showing only minor variation (128.90 to 121.88 nm) and zeta potential changing slightly (−41.90 to −39.96 mV). However, when the centrifugal speed exceeded 4000 rpm, more pronounced changes were observed: particle size decreased to 89.85 nm, and zeta potential shifted to −26.05 mV. Notably, even after centrifugation at 10 000 rpm, the zeta potential remained at −26.05 mV.

### 3.5 Bioactivity

#### 3.5.1 Contact Toxicity

**FIGURE 6:**
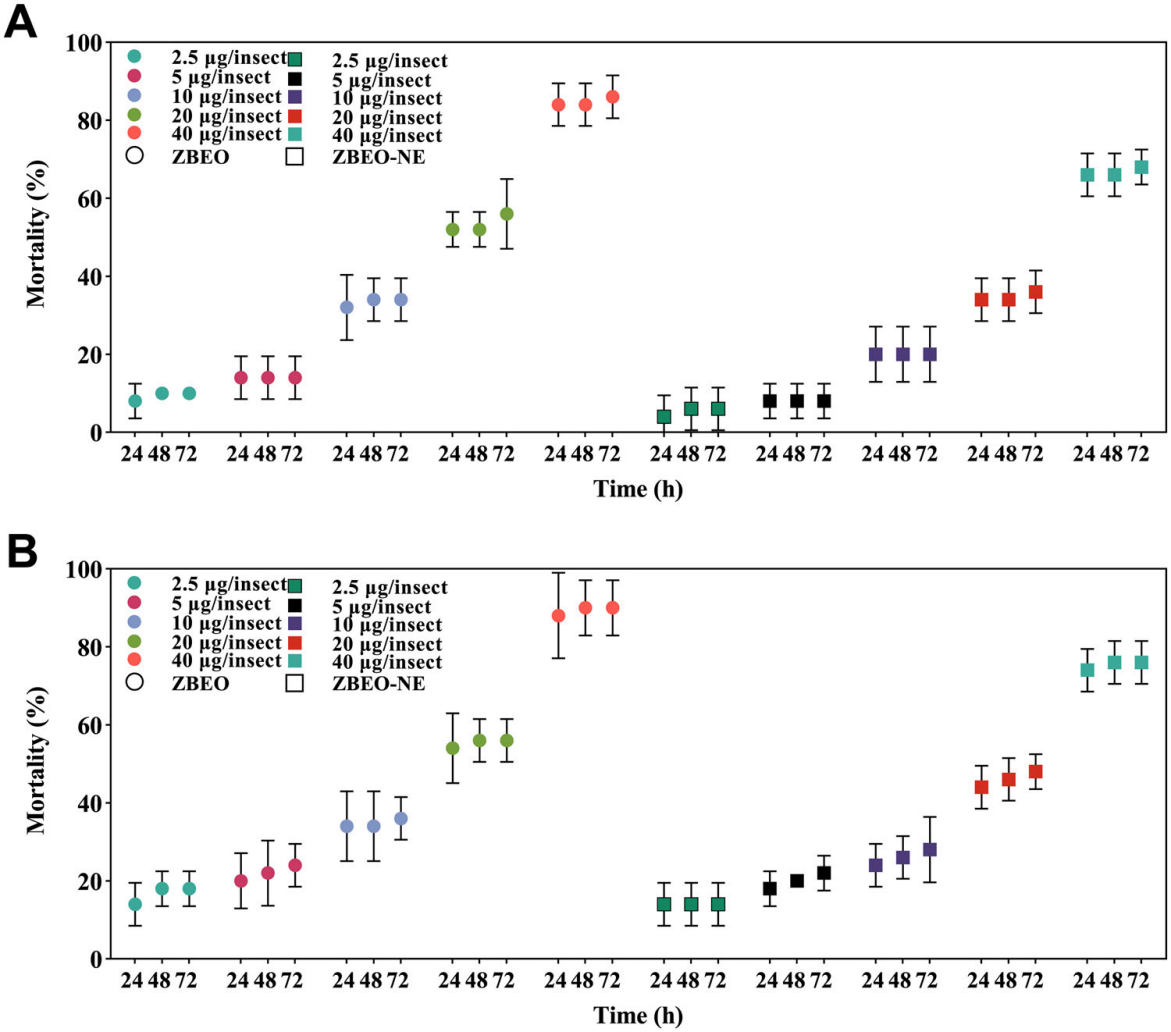
Contact toxicity of ZBEO and ZBEO‐NE against *T. castaneum* adults and larvae. (A) Adults; (B) larvae. Values are presented as means ± SD (*n* = 5).

The contact toxicities of ZBEO and ZBEO‐NE against *T. castaneum* adults and larvae were evaluated at 24, 48, and 72 h. The corresponding LD_50_ and LD_90_ values are presented in Table 5, whereas the mortality data are shown in Figure 6. For adults treated with ZBEO, the LD_50_ values were 16.03, 15.66, and 14.56 µg/insect at 24, 48, and 72 h, respectively, compared with 13.79, 12.72, and 12.33 µg/insect for larvae. The corresponding LD_50_ values of ZBEO‐NE were 27.83, 28.26, and 26.62 µg/insect for adults and 20.77, 19.02, and 18.03 µg/insect for larvae. Thus, larvae exhibited lower LD_50_ values than adults for both formulations at all exposure times, indicating greater susceptibility at the median‐effect level. However, differences in LD_90_ values between the two life stages were less consistent. ZBEO showed lower LD_50_ and LD_90_ values than ZBEO‐NE in both adults and larvae, indicating greater contact toxicity. Mortality increased with increasing dose for both formulations and life stages (Figure 6). Neither acetone nor the blank NE caused observable mortality, whereas pyrethrins exhibited substantially greater contact toxicity than ZBEO and ZBEO‐NE.

**TABLE 5:** LD_50_ and LD_90_ values of ZBEO and ZBEO‐NE against *T. castaneum* adults and larvae in the contact toxicity assay.

Treatment	Time (h)	Insect	LD_50_ (µg/insect) (95% CI)	LD_90_ (µg/insect) (95% CI)	Slope ± SE	*χ* ^2^	*P*
ZBEO	24	Adult	16.03 (13.05–20.35)	69.67 (47.61–125.01)	2.01 ± 0.24	7.23	0.99
		Larva	13.79 (11.50–16.87)	71.16 (49.92–118.78)	1.80 ± 0.22	8.46	0.99
	48	Adult	15.66 (12.65–20.04)	72.83 (48.82–135.49)	1.92 ± 0.24	5.66	0.99
		Larva	12.72 (10.56–15.57)	70.79 (49.16–120.19)	1.72 ± 0.20	5.24	0.99
	72	Adult	14.56 (11.84–18.39)	64.84 (44.49–115.34)	1.98 ± 0.23	6.17	0.99
		Larva	12.33 (10.42–14.76)	70.61 (50.48–112.95)	1.69 ± 0.17	8.08	0.99
ZBEO‐NE	24	Adult	27.83 (21.56–39.84)	137.07 (81.30–331.46)	1.85 ± 0.26	9.41	0.99
		Larva	20.77 (17.21–26.13)	159.66 (102.60–300.61)	1.45 ± 0.21	8.65	0.99
	48	Adult	28.26 (21.54–41.67)	154.51 (87.76–404.14)	1.74 ± 0.25	9.43	0.99
		Larva	19.02 (16.07–23.21)	141.72 (95.40–245.20)	1.47 ± 0.20	7.50	0.98
	72	Adult	26.62 (20.57–38.09)	137.56 (80.99–335.89)	1.78 ± 0.25	9.44	0.99
		Larva	18.03 (15.27–21.89)	137.37 (92.83–236.17)	1.45 ± 0.24	7.15	0.99
Pyrethrins	24	Adult	0.11 (0.09–0.13)	0.40 (0.29–0.65)	2.02 ± 0.23	7.48	0.99
		Larva	0.07 (0.05–0.08)	0.22 (0.17–0.32)	2.43 ± 0.27	10.59	0.98
	48	Adult	0.08 (0.07–0.10)	0.37 (0.27–0.61)	1.94 ± 0.23	10.42	0.98
		Larva	0.06 (0.05–0.07)	0.21 (0.16–0.30)	2.40 ± 0.27	12.85	0.96
	72	Adult	0.08 (0.06–0.09)	0.32 (0.24–0.51)	2.05 ± 0.24	9.13	0.99
		Larva	0.06 (0.05–0.07)	0.20 (0.16–0.29)	2.40 ± 0.28	11.96	0.97
Linalool^a^	24	Adult	17.06 (14.84–28.74)	—	2.52 ± 0.46	17.89	0.22
		Larva	16.42 (12.99–21.46)	—	2.80 ± 0.47	23.24	0.72

The LD_50_ and LD_90_ of ZBEO and ZBEO‐NE against *T. castaneum* adults and larvae were determined by probit analysis (*n* = 5).

^a^Data cited from the research by [20].

#### 3.5.2 Fumigant Toxicity

**FIGURE 7:**
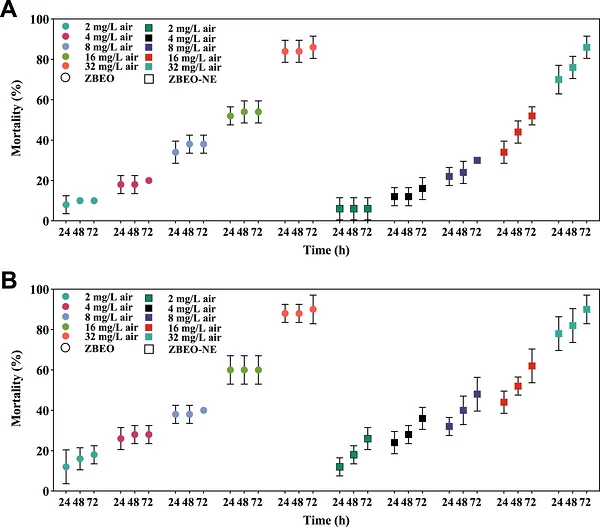
Fumigant toxicity of ZBEO and ZBEO‐NE against T. castaneum adults and larvae. (A) Adults; (B) larvae. Values are presented as means ± SD (*n* = 5).

The fumigant toxicities of ZBEO and ZBEO‐NE against *T. castaneum* adults and larvae were evaluated over a 72‐h exposure period. As summarized in Table 6, ZBEO exhibited relatively stable LC_50_ values of 12.34, 11.63, and 11.21 mg/L air in adults and 9.84, 9.41, and 8.92 mg/L air in larvae at 24, 48, and 72 h, respectively. In contrast, the fumigant toxicity of ZBEO‐NE increased markedly with exposure time, with LC_50_ values decreasing from 20.62 to 12.68 mg/L air in adults and from 14.05 to 7.06 mg/L air in larvae. Larvae consistently exhibited lower LC_50_ values than adults for both formulations, indicating greater susceptibility at the median‐effect level. The LC_90_ values of ZBEO remained relatively stable, whereas those of ZBEO‐NE generally decreased with prolonged exposure, although differences between adults and larvae were not fully consistent. At 24 and 48 h, ZBEO generally exhibited greater fumigant toxicity than ZBEO‐NE. By 72 h, however, ZBEO‐NE approached or exceeded ZBEO for some toxicity endpoints, including the LC_90_ in adults and the LC_50_ in larvae. Mortality increased with increasing concentration for both formulations, while the increase over time was more pronounced for ZBEO‐NE, particularly at the higher concentrations (Figure 7). Neither acetone nor the blank NE caused observable mortality, whereas phosphine exhibited substantially greater fumigant toxicity than both ZBEO and ZBEO‐NE.

**TABLE 6:** LC_50_ and LC_90_ values of ZBEO and ZBEO‐NE against *T. castaneum* adults and larvae in the fumigant toxicity assay.

Treatment	Time (h)	Insect	LC_50_ (mg/L air) (95% CI)	LC_90_ (mg/L air) (95% CI)	Slope ± SE	*χ* ^2^	*P*
ZBEO	24	Adult	12.34 (9.98–15.75)	57.20 (38.45–106.03)	1.93 ± 0.24	5.49	0.99
		Larva	9.84 (8.60–11.33)	49.14 (37.82–69.24)	1.84 ± 0.22	9.80	0.99
	48	Adult	11.63 (9.36–14.88)	56.93 (37.90–107.52)	1.86 ± 0.23	3.96	0.99
		Larva	9.41 (8.24–10.81)	54.01 (41.26–76.70)	1.69 ± 0.25	7.72	0.99
	72	Adult	11.21 (9.04–14.27)	54.09 (36.32–100.44)	1.87 ± 0.23	4.06	0.99
		Larva	8.92 (7.72–10.36)	51.47 (38.64–75.41)	1.68 ± 0.20	7.84	0.98
ZBEO‐NE	24	Adult	20.62 (15.81–29.91)	116.03 (66.43–300.39)	1.71 ± 0.24	8.77	0.99
		Larva	14.05 (11.89–17.07)	103.15 (69.86–177.42)	1.48 ± 0.26	6.79	0.98
	48	Adult	16.54 (13.20–21.98)	77.32 (49.61–157.34)	1.91 ± 0.25	7.54	0.99
		Larva	11.22 (9.40–13.67)	89.67 (59.67–160.31)	1.42 ± 0.23	7.01	0.99
	72	Adult	12.68 (10.40–15.89)	51.06 (35.78–87.52)	2.12 ± 0.25	7.43	0.99
		Larva	7.06 (5.86–8.46)	55.85 (28.82–93.88)	1.43 ± 0.21	9.27	0.99
Phosphine	24	Adult	0.94 (0.78–1.15)	3.14 (2.32–4.86)	2.21 ± 0.24	9.34	0.99
		Larva	0.58 (0.47–0.71)	2.38 (1.77–3.69)	2.09 ± 0.24	9.12	0.99
	48	Adult	0.86 (0.71–1.05)	2.96 (2.18–4.62)	2.15 ± 0.24	10.25	0.99
		Larva	0.53 (0.43–0.64)	2.02 (1.53–3.04)	2.20 ± 0.26	9.52	0.99
	72	Adult	0.81 (0.67–0.99)	2.81 (2.08–4.39)	2.14 ± 0.24	9.70	0.99
		Larva	0.50 (0.41–0.60)	1.81 (1.39–2.66)	2.30 ± 0.27	10.10	0.99
Linalool^a^	24	Adult	15.82 (13.29–17.93)	—	4.32 ± 0.44	15.58	0.92
		Larva	16.73 (14.57–18.14)	—	4.68 ± 0.49	10.27	0.87

The LC_50_ and LC_90_ of ZBEO and ZBEO‐NE against *T. castaneum* adults and larvae were determined by probit analysis (*n* = 5).

^a^ Data cited from the research by [20].

#### 3.5.3 Repellency Activity

The repellent behavioral responses of *T. castaneum* adults to ZBEO and ZBEO‐NE were evaluated across different concentration gradients at observation times of 2, 4, 6, 12, 24, 48, and 72 h. Behavioral differences were statistically analyzed using chi‐square tests (Figure 8). Both ZBEO and ZBEO‐NE exerted significant repellent effects during the early observation period, exhibiting a clear concentration‐dependent relationship. At the highest concentration, complete repellency was observed within the initial 2 and 4 h. Although the repellent activity gradually decreased after 4 h, significant repellency was still maintained at 72 h under high‐dose treatments. In contrast, the low‐concentration groups gradually lost statistically significant repellent activity by 48 h. Across all observation times and tested concentrations, ZBEO‐NE consistently exhibited more stable and stronger repellent effects than raw ZBEO. Furthermore, chi‐square analysis confirmed that ZBEO‐NE retained significant repellent activity for up to 72 h at relatively high doses, whereas the repellency of unformulated ZBEO gradually reduced over time.

FIGURE 8Preference rate of *T. castaneum* adults towards ZBEO and ZBEO‐NE at different concentrations under various treatment durations (A: 2 h, B: 4 h, C: 6 h, D: 12 h, E: 24 h, F: 48 h, G: 72 h). The left and right bars indicate the treated and control semicircles, respectively. Chi‑square test, *n* = 5. ns: *p* > 0.05; *: *p* < 0.05; **: *p* < 0.01; ***: *p* < 0.001.

**Figure:**
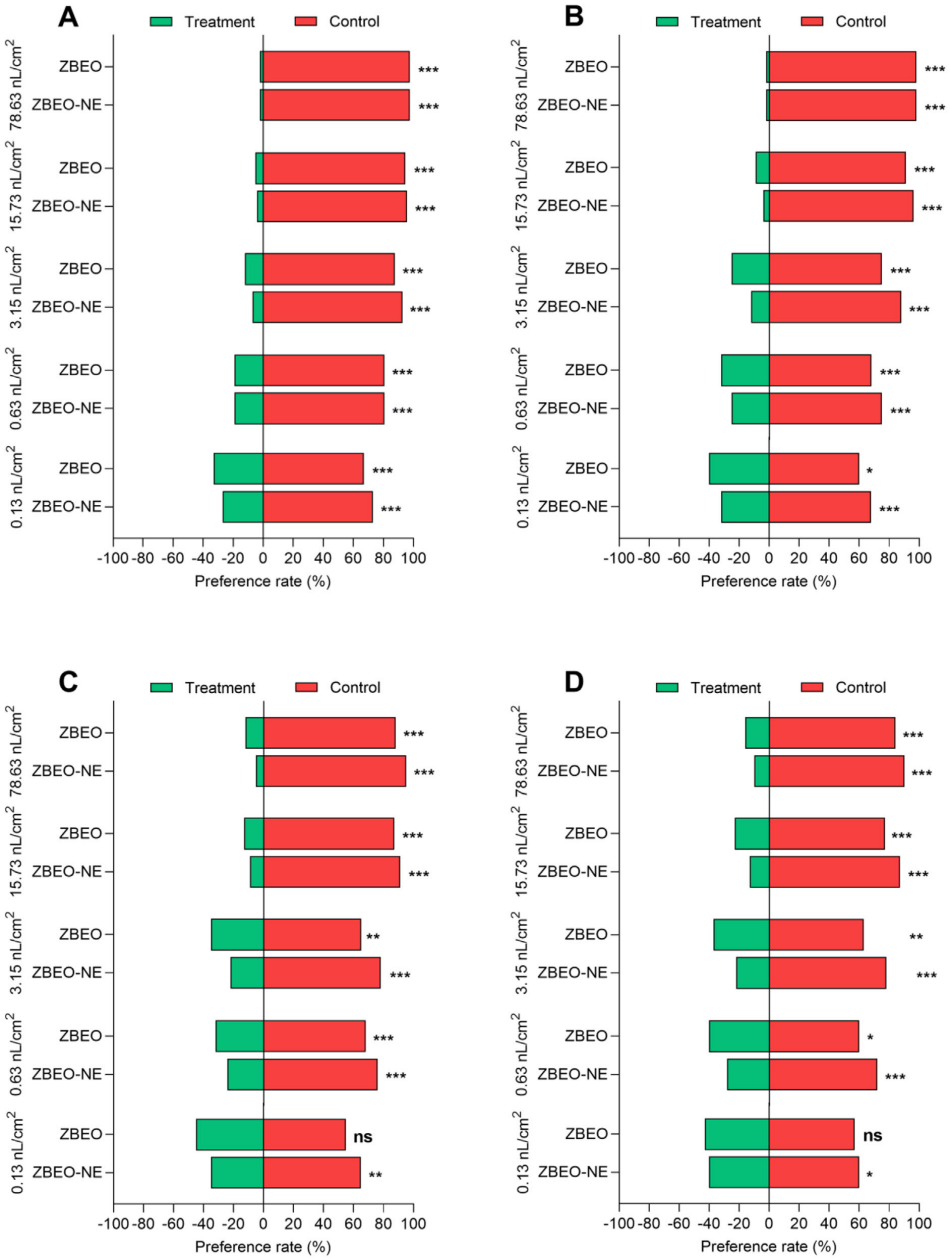

**Figure:**
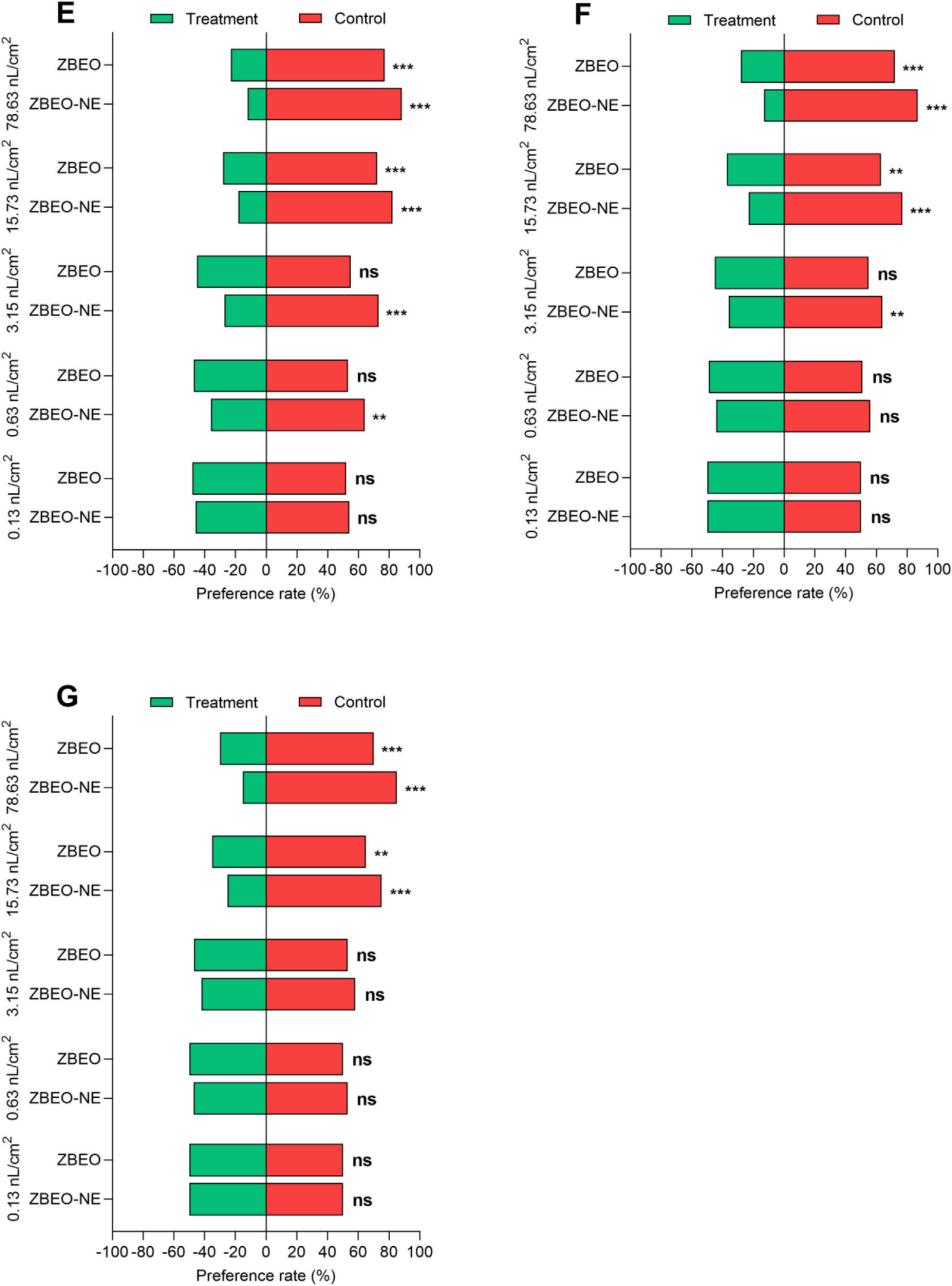

### 3.6 Retention of Biological Activity After Stability Treatments

**FIGURE 9:**
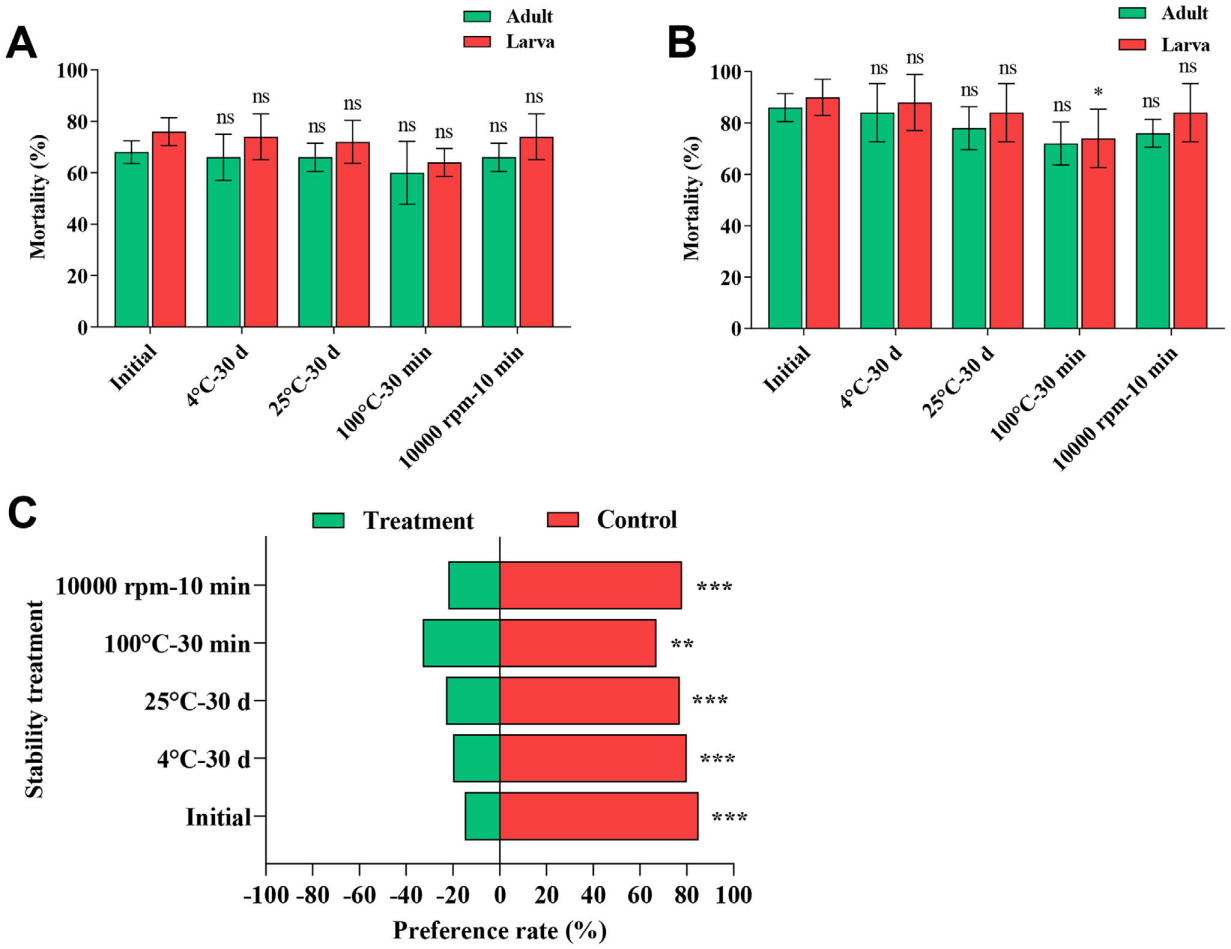
Biological activity of ZBEO‐NE after stability treatments. (A) Contact mortality against *T. castaneum* adults and larvae; (B) fumigant mortality against adults and larvae; and (C) repellency against adults at 72 h. In (A) and (B), significance was determined relative to the initial sample within each life stage. Values are presented as means ± SD (*n* = 5). ns: *p* > 0.05; *: *p* < 0.05. In (C), asterisks indicate significant deviations from the theoretical 50:50 distribution by Chi‐square tests. **, *p* < 0.01; ***, *p* < 0.001.

ZBEO‐NE retained substantial biological activity after storage, heating, and centrifugation treatments (Figure 9). Contact mortality against both adults and larvae remained comparable to that of the initial sample, with no significant differences observed after storage at 4°C or 25°C for 30 days, heating at 100°C for 30 min, or centrifugation at 10 000 rpm for 10 min (Figure 9A). Fumigant mortality was also largely maintained following these treatments, although heating at 100°C for 30 min significantly reduced mortality in adults, while no significant changes were detected in larvae (Figure 9B). In the two‐choice assay, the distribution of adults for all samples deviated significantly from the theoretical 50:50 distribution, confirming that ZBEO‐NE retained significant repellency after storage, heating, and centrifugation treatments (Figure 9C). Overall, the formulation maintained good contact, fumigant, and repellent activities under the tested stability conditions.

### 3.7 Cytotoxicity of ZBEO‐NE Toward L‐929 Cells

**FIGURE 10:**
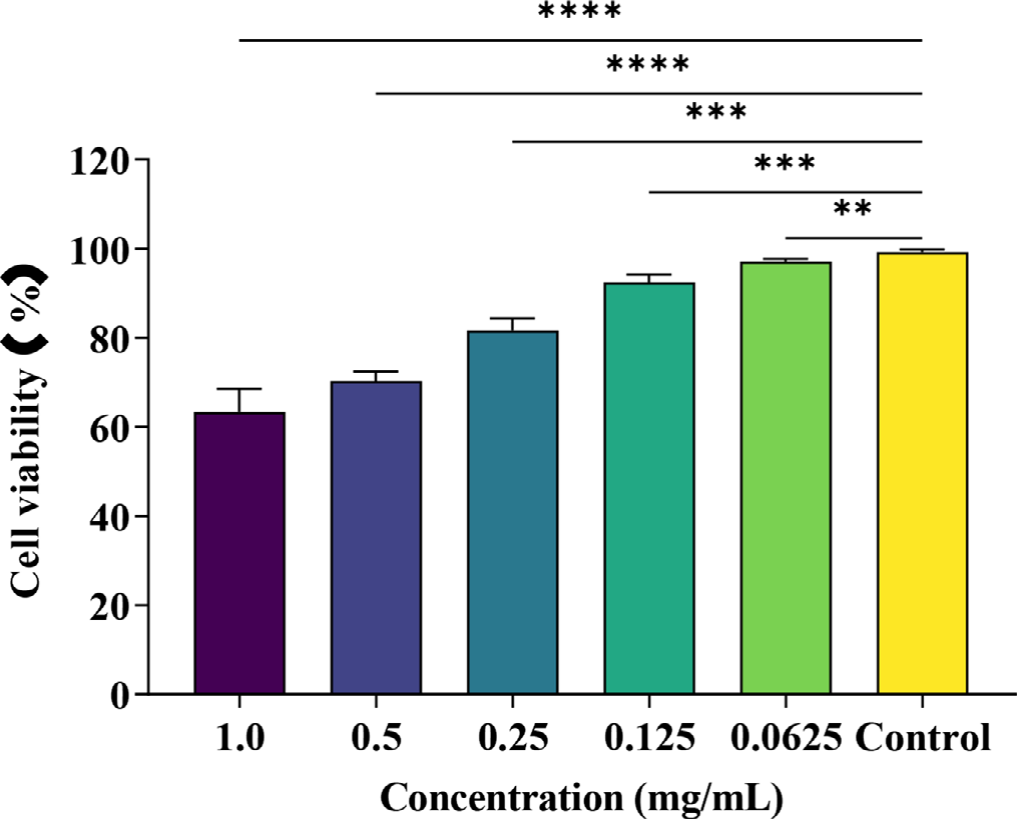
Cytotoxicity of ZBEO‐NE toward L‐929 cells. Values are presented as means ± SD (*n* = 5). Asterisks indicate significant differences compared with the control group: **, *p* < 0.01; ***, *p* < 0.001; ****, *p* < 0.0001.

ZBEO‐NE affected L‐929 cell viability in a concentration‐dependent manner (Figure 10). Compared with the control group, significant reductions in cell viability were observed at all tested concentrations, with the greatest reduction occurring at the highest concentration. However, cell viability remained above 80% at concentrations of 0.25 mg/mL or lower and exceeded 90% at 0.125 mg/mL or lower. These findings indicate that the cytotoxicity of ZBEO‐NE decreased markedly with dilution and that the formulation exhibited good cytocompatibility at relatively low concentrations.

## 4 Discussion

The EO yield obtained from *Z. bungeanum* in this study was 4.88%, exceeding the 2.90% reported previously using steam distillation [32]. Such variation may reflect differences in cultivar, growing conditions, harvest time, and extraction method [33]. GC–MS analysis identified linalool (53.78%) as the predominant component, substantially higher than the 21.76% reported in our previous study [20]. A different chemical profile was also observed compared with *Z. bungeanum* collected from the same region, in which limonene (24.71%) and linalool (23.13%) occurred at comparable proportions [34]. These findings confirm that the yield and volatile composition of ZBEO can vary considerably with genetic and environmental factors [35, 36].

The prepared ZBEO‐NE exhibited favorable colloidal properties, with a mean droplet size of 128.90 nm, a PDI of 0.20, and a zeta potential of −41.90 mV. These values indicate a nanoscale, relatively uniform, and electrostatically stable dispersion [18, 37, 38]. The higher absolute zeta potential compared with the Tween‐80–stabilized system reported previously [14] may be related to the mixed Tween‐80/Span‐80 interfacial layer and the high‐energy emulsification process. Probe ultrasonication likely promoted droplet disruption and reduced size heterogeneity, thereby improving physical stability. The small and uniformly distributed droplets may also enhance dispersion, surface coverage, and consistency of bioactive compound delivery, while the high absolute zeta potential helps limit aggregation during storage.

TEM showed predominantly spherical droplets with diameters of approximately 100 nm, slightly smaller than the hydrodynamic diameter measured by DLS. This difference is expected because TEM examines dried droplets, whereas DLS includes the interfacial layer and associated solvent molecules in dispersion [1]. No obvious aggregation or coalescence was observed, supporting the uniform structure of ZBEO‐NE. The formulation also exhibited shear‐thinning behavior, with viscosity decreasing above a shear rate of approximately 2 s^−1^, consistent with related NE systems [39, 40, 41]. This behavior may result from the alignment and weakening of droplet interactions under shear. Such flow characteristics can facilitate pumping, spraying, and surface spreading while allowing the formulation to recover a higher apparent viscosity at low shear, thereby supporting its practical application and retention on treated surfaces.

ZBEO‐NE maintained good physical stability during 30 days of storage at both 4°C and 25°C, with no phase separation and only minor changes in droplet size, PDI, and zeta potential. This performance was superior to that of the carboxymethyl chitosan/Tween‐80–stabilized garlic oil NE, which exhibited phase separation within the same period [42]. The consistently low PDI and strongly negative zeta potential indicate that the interfacial layer and electrostatic repulsion effectively limited droplet aggregation and coalescence. The slight decrease in particle size may be attributed to the settling of a small fraction of larger droplets [43]. These findings are consistent with previous observations for cinnamon EO‐NEs [26] and highlight the importance of surfactant selection and optimized preparation conditions in maintaining the physical stability of EO‐NEs.

Thermal and centrifugal stability tests were used to assess the resistance of ZBEO‐NE to temperature fluctuations and mechanical stress during processing, transportation, and storage [17]. ZBEO‐NE remained physically stable under the tested temperatures, consistent with findings for *Myristica fragrans* EO‐NEs [44]. This stability may be associated with the protective interfacial film formed by the mixed surfactant system. Under centrifugation below 4000 rpm, only minor changes in droplet size and zeta potential were observed. At higher speeds, droplet size decreased, and the absolute zeta potential was reduced, possibly because of the selective redistribution of larger droplets and changes in the interfacial organization [45]. Nevertheless, no phase separation was observed even at 10 000 rpm, indicating that the formulation retained acceptable physical integrity under strong mechanical stress.

The insecticidal activity of ZBEO and ZBEO‐NE was evaluated against both adults and larvae of *T. castaneum*. The reduced immediate contact toxicity observed after nanoencapsulation was consistent with the findings of Santos et al. [46] and likely reflected differences in delivery mechanisms and bioavailability. Monoterpenoids, the principal bioactive constituents of ZBEO, are volatile and lipophilic compounds capable of penetrating the insect cuticle and interacting with key molecular targets involved in neural and metabolic functions, such as acetylcholinesterase and arginine kinase [47]. Although nanoscale droplets can improve dispersion, surface coverage, and contact with the insect cuticle, the active compounds incorporated in ZBEO‐NE must first partition from the surfactant‐stabilized droplets before becoming available for cuticular penetration. In contrast, unencapsulated ZBEO allows more immediate contact between free lipophilic constituents and the insect surface. This delayed availability, together with the interfacial barrier provided by the surfactant layer, may therefore explain the higher LD_50_ and LD_90_ values observed for ZBEO‐NE in the contact toxicity assays [48]. The stronger contact toxicity of the present ZBEO sample than that reported previously [20] may be associated with its different chemical profile. Linalool was the predominant constituent and is known to possess insecticidal activity; however, other abundant components, particularly limonene and linalyl acetate, may also contribute through additive or synergistic effects [20]. These findings highlight the strong influence of the relative abundance of key bioactive constituents on the insecticidal efficacy of EOs [49].

In contrast, the fumigant activity of ZBEO‐NE increased progressively with exposure time, as reflected by the decreasing LC_50_ and LC_90_ values. Free ZBEO can rapidly generate bioactive vapors, whereas incorporation into NE droplets may moderate the loss of volatile constituents and allow their gradual transfer into the headspace [1]. Similar behavior has been reported previously, indicating that NE formulations can provide prolonged release while maintaining relatively stable lethal dose values over time [50, 51]. Once inhaled through the spiracles, monoterpenoids can reach internal target sites and potentially interfere with neural and metabolic processes involved in insect survival [45, 52]. This difference in exposure route provides a plausible explanation for the contrasting contact and fumigant responses of ZBEO‐NE. Nevertheless, because release kinetics were not directly measured, the prolonged fumigant effect should be interpreted as being consistent with gradual volatile release rather than as direct evidence of a sustained‐release mechanism.

Repellency assays showed that both ZBEO and ZBEO‐NE produced strong initial repellent effects, likely due to volatile monoterpenoids interacting with the insect olfactory system [10, 53]. Compared with free ZBEO, ZBEO‐NE maintained repellency for a longer period, suggesting that nanoemulsification may help reduce the rapid loss of volatile constituents and prolong their presence in the test environment [54, 55]. This effect may allow repellent compounds to remain available for olfactory stimulation over an extended period. Thus, nanoemulsification appears to improve the persistence of ZBEO repellency, although direct release‐kinetics measurements would be required to confirm the underlying release behavior.

The retention of biological activity after stability treatments further supports the practical stability of ZBEO‐NE. Preservation of relatively small and uniformly dispersed droplets may help maintain contact with the insect cuticle and facilitate the delivery of lipophilic compounds, whereas substantial droplet growth or loss of interfacial stability could adversely affect biological performance [56]. Contact toxicity against both adults and larvae remained comparable to the initial sample after storage at 4 and 25°C, heating, and centrifugation, indicating that these treatments did not substantially impair its contact activity. Similar studies have shown that EO‐NEs can maintain physicochemical stability while preserving insecticidal efficacy against stored‐product pests [57, 58, 59]. Fumigant activity was also largely retained, although heating at 100°C significantly reduced mortality in adults, possibly because thermal exposure altered the retention or volatilization of active constituents. In contrast, centrifugation caused no marked loss of biological efficacy, consistent with the maintenance of colloidal integrity. Moreover, insect distributions in the two‐choice assay remained significantly different from the theoretical 50:50 distribution after all stability treatments, confirming the persistence of repellency. Overall, the limited physicochemical changes were accompanied by generally preserved insecticidal and repellent activities, supporting a relationship between colloidal stability and functional performance of ZBEO‐NE.

The cytotoxicity assay provided a preliminary assessment of the safety of ZBEO‐NE toward non‐target mammalian cells. ZBEO‐NE showed concentration‐dependent cytotoxicity toward L‐929 fibroblasts, while relatively low concentrations exhibited good cytocompatibility, suggesting that its safety is strongly dose‐dependent. However, an in vitro fibroblast model cannot fully represent the responses of non‐target organisms, and further ecotoxicological and in vivo evaluations are required before practical application [60]. In addition, the cost‐effectiveness of high‐energy emulsification should be improved for large‐scale production [61], and the environmental fate of surfactants and regulatory requirements for nanopesticides require further consideration [62]. Future studies should therefore include large‐scale warehouse simulations and validation under real‐world grain‐storage conditions to confirm the efficacy and safety of ZBEO‐NE in practice.

## 5 Conclusion

A stable ZBEO‐NE with favorable physicochemical properties was successfully developed and evaluated against both adults and larvae of *T. castaneum*. The optimized formulation showed nanoscale droplet size, good colloidal stability, and shear‐thinning behavior. Although ZBEO‐NE exhibited lower contact toxicity than unencapsulated ZBEO, its fumigant activity increased with exposure time, and its repellent effect was more persistent. Importantly, the formulation largely retained its contact, fumigant, and repellent activities after storage, heating, and centrifugation, indicating good functional stability. Cytotoxicity testing using L‐929 cells further showed that ZBEO‐NE exhibited good cytocompatibility at relatively low concentrations. Overall, nanoemulsification improved the physical stability and persistence of ZBEO while maintaining substantial insecticidal and repellent activities, supporting its potential application in stored‐product pest management.

## Author Contributions

**Xu‐Dong Wang**: conceptualization, methodology, investigation, writing – original draft. **Zhi‐Qin Liu**: methodology, validation, formal analysis. **Xue Fan**: software, data curation. **Jia‐Li Bai**: resources, visualization. **Jun‐Yu Liang**: writing – review and editing. **Ji Zhang**: supervision, project administration, funding acquisition, writing – review and editing. All authors have read and agreed to the published version of the manuscript.

## Conflicts of Interest

The authors declare that this research was conducted in the absence of any commercial or financial relationships that could be construed as a potential conflicts of interest.

## Data Availability

The data that support the findings of this study are available from the corresponding author upon reasonable request.

## References

[1] A. K. Jasman , A. K. Slomy , and A. I. Khaleel , “Nanoemulsion‐Enhanced Insecticidal Activity of *Cymbopogon Citratus* and *Mentha Longifolia* Essential Oils Against the Red Flour Beetle (*Tribolium castaneum*),” Journal of Stored Products Research 114 (2025): 102759, 10.1016/j.jspr.2025.102759.

[2] T. Zhang , Y.‐J. Wang , W. Guo , et al., “DNA Barcoding, Species‐Specific PCR, and Real‐Time PCR Techniques for the Identification of Six *Tribolium* Pests of Stored Products,” Scientific Reports 6 (2016): 28494, 10.1038/srep28494. PMC4926120 27352804

[3] J. F. Campbell , C. G. Athanassiou , D. W. Hagstrum , and K. Y. Zhu , “Tribolium Castaneum: A Model Insect for Fundamental and Applied Research,” Annual Review of Entomology 67 (2022): 347–365, 10.1146/annurev-ento-080921-075157. 34614365

[4] J. S. Oviedo‐Sarmiento , J. J. B. Cortes , W. A. Delgado Ávila , et al., “Fumigant Toxicity and Biochemical Effects of Selected Essential Oils Toward the Red Flour Beetle, *Tribolium Castaneum* (Coleoptera: Tenebrionidae),” Pesticide Biochemistry and Physiology 179 (2021): 104941, 10.1016/j.pestbp.2021.104941. 34802531

[5] S. Duarte , A. Magro , J. Tomás , et al., “The Interaction Between *Tribolium Castaneum* and Mycotoxigenic *Aspergillus Flavus* in Maize Flour,” Insects 12 (2021): 730, 10.3390/insects12080730. PMC8396807 34442296

[6] D. Moutassem , T. Boubellouta , Y. Bellik , et al., “Insecticidal Activity of *Thymus Pallescens* De Noë and *Cymbogon Citratus* Essential Oils Against *Sitophilus Zeamais* and *Tribolium Castaneum* ,” Scientific Reports 14 (2024): 13951, 10.1038/s41598-024-64757-3. PMC11183130 38886531

[7] G. P. Opit , T. W. Phillips , M. J. Aikins , and M. M. Hasan , “Phosphine Resistance in *Tribolium Castaneum* and *Rhyzopertha dominica* From Stored Wheat in Oklahoma,” Journal of Economic Entomology 105 (2012): 1107–1114, 10.1603/EC12064. 22928286

[8] V. M. Pathak , V. K. Verma , B. S. Rawat , et al., “Present Status of Insecticide Impacts and Eco‐Friendly Approaches for Remediation—A Review,” Environmental Research 240 (2024): 117432, 10.1016/j.envres.2023.117432. 37865327

[9] K. Yang , C. F. Wang , C. X. You , et al., “Bioactivity of Essential Oil of *Litsea Cubeba* From China and Its Main Compounds Against Two Stored Product Insects,” Journal of Asia‐Pacific Entomology 17 (2014): 459–466, 10.1016/j.aspen.2014.03.011.

[10] J.‐Y. Liang , Z.‐B. Hou , H.‐S. Wu , et al., “Chemical Constituents of Essential Oil Extracted From *Rhododendron Anthopogonoides* and Its Bioactivities Against *Tribolium Castaneum* and *Ditylenchus Destructor* ,” Biochemical Systematics and Ecology 103 (2022): 104431, 10.1016/j.bse.2022.104431.

[11] C. Regnault‐Roger , C. Vincent , and J. T. Arnason , “Essential Oils in Insect Control: Low‐Risk Products in a High‐Stakes World,” Annual Review of Entomology 57 (2012): 405–424, 10.1146/annurev-ento-120710-100554. 21942843

[12] Q. Yang , F. Zheng , Q. Chai , et al., “Effect of Emulsifiers on the Properties of Corn Starch Films Incorporated With *Zanthoxylum Bungeanum* Essential Oil,” International Journal of Biological Macromolecules 256 (2024): 128382, 10.1016/j.ijbiomac.2023.128382. 38000598

[13] Y. Bao , L. Yang , Q. Fu , et al., “The Current Situation of *Zanthoxylum Bungeanum* Industry and the Research and Application Prospect. A Review,” Fitoterapia 164 (2023): 105380, 10.1016/j.fitote.2022.105380. 36462661

[14] H. Zheng , S. Chen , Z. Liu , et al., “Utilization of Nanoemulsion to Enhance Antibacterial Capacity of *Zanthoxylum Bungeanum* Pericarp Essential Oils Against Foodborne Pathogenic Bacteria,” LWT 207 (2024): 116657, 10.1016/j.lwt.2024.116657.

[15] K. Sarmah , T. Anbalagan , M. Marimuthu , P. Mariappan , S. Angappan , and S. Vaithiyanathan , “Innovative Formulation Strategies for Botanical‐ and Essential Oil‐Based Insecticides,” Journal of Pest Science 98 (2025): 1–30, 10.1007/s10340-024-01846-2.

[16] H. Falleh , “Demystifying the Power of Essential Oils: A Review of Their Antibacterial Properties and Potential as Natural Food Preservatives,” EXCLI Journal 24 (2025): 828–850, 10.17179/excli2025-8439. PMC12419452 40933954

[17] N. Dasgupta , S. Ranjan , and M. Gandhi , “Nanoemulsion Ingredients and Components,” Environmental Chemistry Letters 17 (2019): 917–928, 10.1007/s10311-018-00849-7.

[18] B. D. da Silva , D. K. A. do Rosário , D. A. Weitz , and C. A. Conte‐Junior , “Essential Oil Nanoemulsions: Properties, Development, and Application in Meat and Meat Products,” Trends in Food Science & Technology 121 (2022): 1–13, 10.1016/j.tifs.2022.01.026.

[19] S. Kumar , M. Nehra , N. Dilbaghi , G. Marrazza , A. A. Hassan , and K.‐H. Kim , “Nano‐Based Smart Pesticide Formulations: Emerging Opportunities for Agriculture,” Journal of Controlled Release 294 (2019): 131–153, 10.1016/j.jconrel.2018.12.012. 30552953

[20] J.‐Y. Liang , Y. An , Z.‐B. Hou , et al., “Acute Toxicity of Zanthoxylum Bungeanum Against Two Stored Product Insects and Synergistic Interactions Between Two Major Compounds Limonene and Linalool,” Journal of Environmental Science and Health, Part B 57 (2022): 739–744, 10.1080/03601234.2022.2107370. 35930275

[21] X.‐D. Wang , J.‐L. Bai , X. Fan , Z.‐Q. Liu , and J. Zhang , “Anti‐Inflammatory, Antioxidant, and Antibacterial Activity of Tea Saponin‐Based *Zanthoxylum Bungeanum* Essential Oil Nanoemulsions Against Bovine Mastitis Pathogens,” Industrial Crops and Products 237 (2025): 122241, 10.1016/j.indcrop.2025.122241.

[22] Y. Chu , W. Cheng , X. Feng , et al., “Fabrication, Structure, and Properties of Pullulan‐Based Active Films Incorporated With Ultrasound‐Assisted Cinnamon Essential Oil Nanoemulsions,” Food Packaging and Shelf Life 25 (2020): 100547, 10.1016/j.fpsl.2020.100547.

[23] J. Wan , S. Zhong , P. Schwarz , B. Chen , and J. Rao , “Physical Properties, Antifungal, and Mycotoxin Inhibitory Activities of Five Essential Oil Nanoemulsions: Impact of Oil Compositions and Processing Parameters,” Food Chemistry 291 (2019): 199–206, 10.1016/j.foodchem.2019.04.032. 31006459

[24] S. Wang , X. Wang , M. Liu , et al., “Preparation and Characterization of *Eucommia Ulmoides* Seed Oil O/W Nanoemulsion by Dynamic High‐Pressure Microfluidization,” LWT 121 (2020): 108960, 10.1016/j.lwt.2019.108960.

[25] M. Chang , Y. Guo , Z. Jiang , et al., “Sea Buckthorn Pulp Oil Nanoemulsions Fabricated by Ultra‐High Pressure Homogenization Process: A Promising Carrier for Nutraceutical,” Journal of Food Engineering 287 (2020): 110129, 10.1016/j.jfoodeng.2020.110129.

[26] P. Pongsumpun , S. Iwamoto , and U. Siripatrawan , “Response Surface Methodology for Optimization of Cinnamon Essential Oil Nanoemulsion With Improved Stability and Antifungal Activity,” Ultrasonics Sonochemistry 60 (2020): 104604, 10.1016/j.ultsonch.2019.05.021. 31539730

[27] T. M. R. Lotfy , S. M. S. Shawir , and M. E. I. Badawy , “The Impacts of Chitosan‐Essential Oil Nanoemulsions on the Microbial Diversity and Chemical Composition of Refrigerated Minced Meat,” International Journal of Biological Macromolecules 239 (2023): 124237, 10.1016/j.ijbiomac.2023.124237. 37003382

[28] D. Liang , C. Wang , X. Luo , Z. Wang , F. Kong , and Y. Bi , “Preparation, Characterization and Properties of Cinnamon Essential Oil Nano‐Emulsion Formed by Different Emulsifiers,” Journal of Drug Delivery Science and Technology 86 (2023): 104638, 10.1016/j.jddst.2023.104638.

[29] Z. L. Liu and S. H. Ho , “Bioactivity of the Essential Oil Extracted From *Evodia Rutaecarpa* Hook f. et Thomas Against the Grain Storage Insects, *Sitophilus Zeamais* Motsch. and *Tribolium Castaneum* (Herbst),” Journal of Stored Products Research 35 (1999): 317–328, 10.1016/S0022-474X(99)00015-6.

[30] J. S. Zhang , N. N. Zhao , Q. Z. Liu , et al., “Repellent Constituents of Essential Oil of *Cymbopogon Distans* Aerial Parts Against Two Stored‐Product Insects,” Journal of Agricultural and Food Chemistry 59 (2011): 9910–9915, 10.1021/jf202266n. 21819085

[31] N. Flórez‐Fernández , E. Falqué , H. Domínguez , and M. D. Torres , “Green Extraction of Carrageenans From *Mastocarpus Stellatus* ,” Polymers 14 (2022): 554, 10.3390/polym14030554. PMC8839242 35160543

[32] Y. Lan , Q. Wu , Y.‐Q. Mao , et al., “Cytotoxicity and Enhancement Activity of Essential Oil From *Zanthoxylum Bungeanum* Maxim. As a Natural Transdermal Penetration Enhancer,” Journal of Zhejiang University SCIENCE B 15 (2014): 153–164, 10.1631/jzus.B1300230. PMC3924391 24510708

[33] V. P. Katekar , A. B. Rao , and V. R. Sardeshpande , “Review of the Rose Essential Oil Extraction by Hydrodistillation: An Investigation for the Optimum Operating Condition for Maximum Yield,” Sustainable Chemistry and Pharmacy 29 (2022): 100783, 10.1016/j.scp.2022.100783.

[34] Y. Wu , Z. Zhuo , Q. Qian , and D. Xu , “Chemotaxonomic Variation of Volatile Components in *Zanthoxylum Bungeanum* Peel and Effects of Climate on Volatile Components,” BMC Plant Biology 24 (2024): 793, 10.1186/s12870-024-05485-8. PMC11340169 39169301

[35] M. M. Freire , G. N. Jham , O. D. Dhingra , C. M. Jardim , R. C. Barcelos , and V. M. M. Valente , “Composition, Antifungal Activity, and Main Fungitoxic Components of the Essential Oil of Mentha Piperita l,” Journal of Food Safety 32 (2012): 29–36, 10.1111/j.1745-4565.2011.00341.x.

[36] M. Hasanuzzaman , Plant Ecophysiology and Adaptation Under Climate Change: Mechanisms and Perspectives I: General Consequences and Plant Responses (Springer, 2020), 10.1007/978-981-15-2156-0.

[37] A. Prakash , R. Baskaran , N. Paramasivam , and V. Vadivel , “Essential Oil Based Nanoemulsions to Improve the Microbial Quality of Minimally Processed Fruits and Vegetables: A Review,” Food Research International 111 (2018): 509–523, 10.1016/j.foodres.2018.05.066. 30007714

[38] Y. Zhou , S. Sun , W. Bei , M. R. Zahi , Q. Yuan , and H. Liang , “Preparation and Antimicrobial Activity of Oregano Essential Oil Pickering Emulsion Stabilized by Cellulose Nanocrystals,” International Journal of Biological Macromolecules 112 (2018): 7–13, 10.1016/j.ijbiomac.2018.01.102. 29414733

[39] D. Liang , B. Feng , N. Li , et al., “Preparation, Characterization, and Biological Activity of *Cinnamomum Cassia* Essential Oil Nano‐Emulsion,” Ultrasonics Sonochemistry 86 (2022): 106009, 10.1016/j.ultsonch.2022.106009. PMC9058955 35472756

[40] A. Sanchez , M. C. García , M. J. Martín‐Piñero , J. Muñoz , and M.‐C. Alfaro‐Rodríguez , “Elaboration and Characterization of Nanoemulsion With Orange Essential Oil and Pectin,” Journal of the Science of Food and Agriculture 102 (2022): 3543–3550, 10.1002/jsfa.11698. 34854080

[41] M. Sharma , A. K. Singh , D. N. Yadav , S. Arora , and R. K. Vishwakarma , “Impact of Octenyl Succinylation on Rheological, Pasting, Thermal, and Physicochemical Properties of Pearl Millet (*Pennisetum typhoides*) Starch,” LWT 73 (2016): 52–59, 10.1016/j.lwt.2016.05.034.

[42] X. Zhang , Y. Wang , D. Wang , J. Tang , and M. Xu , “Synergistic Stabilization of Garlic Essential Oil Nanoemulsions by Carboxymethyl Chitosan/Tween 80 and Application for Coating Preservation of Chilled Fresh Pork,” International Journal of Biological Macromolecules 266 (2024): 131370, 10.1016/j.ijbiomac.2024.131370. 38580027

[43] S. Zhang , M. Zhang , Z. Fang , and Y. Liu , “Preparation and Characterization of Blended Cloves/Cinnamon Essential Oil Nanoemulsions,” LWT 75 (2017): 316–322, 10.1016/j.lwt.2016.08.046.

[44] L. F. Cossetin , Q. I. Garlet , M. C. Velho , et al., “Development of Nanoemulsions Containing *Lavandula Dentata* or *Myristica Fragrans* Essential Oils: Influence of Temperature and Storage Period on Physical‐Chemical Properties and Chemical Stability,” Industrial Crops and Products 160 (2021): 113115, 10.1016/j.indcrop.2020.113115.

[45] H. Zhang and X. Xia , “Ultrasonic Synergy High‐Speed Shear Improved the Antioxidant and Storage Stability of Clove Essential Oil Nanoemulsion,” Food Bioscience 66 (2025): 106286, 10.1016/j.fbio.2025.106286.

[46] Á. M. O. Santos , A. P. A. Araújo , P. B. Alves , et al., “Characterization and Insecticidal Effects of the Essential Oil and Nanoemulsion of *Pogostemon Cablin* on Populations of *Sitophilus Zeamais* ,” Crop Protection 199 (2026): 107410, 10.1016/j.cropro.2025.107410.

[47] M. A. Seada and A. M. Abouelatta , “Potential for Using Four Plant Essential Oils to Protect Stored Products Against *Tribolium Castaneum* (Herbst) (Coleoptera: Tenebrionidae),” Fine Chemical Engineering 5 (2024): 154–171, 10.37256/fce.5120244391.

[48] D. U. Meenakshi , G. K. Narde , S. Nandhakumar , and A. Ahuja , “Nanoencapsulation as a Strategy to Enhance the Pharmacokinetics of Bioactive Compounds,” Nanotechnologies for Drug Delivery and Biopharmaceutical Development 77 (2025): 253–284, 10.1007/978-3-032-00796-4_9.

[49] J. Yoon and J.‐H. Tak , “Cuticular Property Affects the Insecticidal Synergy of Major Constituents in Thyme Oil Against Houseflies, *Musca Domestica* ,” Scientific Reports 13 (2023): 12654, 10.1038/s41598-023-39898-6. PMC10403520 37542185

[50] M. H. A. El‐Sawah , H. M. Raddy , H. A. Gad , and H. H. Fahmy , “Chemical Composition and Insecticidal Activities of *Origanum Majorana* L. Essential Oil Nanoemulsion Against *Callosobruchus Maculatus* and *Callosobruchus Chinensis* ,” Egyptian Journal of Chemistry 67 (2024): 371–381, 10.21608/ejchem.2024.276355.9445.

[51] H.‐J. Yeom , C.‐S. Jung , J. Kang , et al., “Insecticidal and Acetylcholine Esterase Inhibition Activity of Asteraceae Plant Essential Oils and Their Constituents Against Adults of the German Cockroach (Blattella germanica),” Journal of Agricultural and Food Chemistry 63 (2015): 2241–2248, 10.1021/jf505927n. 25664467

[52] J. M. B. Jemâa , S. Haouel , and M. L. Khouja , “Efficacy of *Eucalyptus* Essential Oils Fumigant Control Against *Ectomyelois Ceratoniae* (Lepidoptera: Pyralidae) Under Various Space Occupation Conditions,” Journal of Stored Products Research 53 (2013): 67–71, 10.1016/j.jspr.2013.02.007.

[53] Q. Gao , L. Song , J. Sun , et al., “Repellent Action and Contact Toxicity Mechanisms of the Essential Oil Extracted From Chinese Chive Against Plutella Xylostella Larvae,” Archives of Insect Biochemistry and Physiology 100 (2019): e21509, 10.1002/arch.21509. 30390324

[54] M. Benjemaa , M. A. Neves , H. Falleh , H. Isoda , R. Ksouri , and M. Nakajima , “Nanoencapsulation of *Thymus capitatus* Essential Oil: Formulation Process, Physical Stability Characterization, and Antibacterial Efficiency Monitoring,” Industrial Crops and Products 113 (2018): 414–421, 10.1016/j.indcrop.2018.01.062.

[55] T. K. Jayasekara , P. C. Stevenson , D. R. Hall , and S. R. Belmain , “Effect of Volatile Constituents From *Securidaca Longepedunculata* on Insect Pests of Stored Grain,” Journal of Chemical Ecology 31 (2005): 303–313, 10.1007/s10886-005-1342-0. 15856785

[56] M. Lokesh , A. Panneerselvam , A. K. Sreekrishnakumar , A. Anand , T. S. Gowda , and S. E. Vendan , “Intensification of Biofumigant Stability: Preparation of Peppermint‐Piperitone Nanoemulsion for Effective Fumigation Against Stored‐Product Beetles,” Journal of Food Science and Technology 63 (2026): 274–283, 10.1007/s13197-024-06181-z. PMC12926266 41737707

[57] T. Adak , N. Barik , N. B. Patil , et al., “Nanoemulsion of Eucalyptus Oil: An Alternative to Synthetic Pesticides Against Two Major Storage Insects (*Sitophilus oryzae* (L.) and *Tribolium Castaneum* (Herbst)) of Rice,” Industrial Crops and Products 143 (2020): 111849, 10.1016/j.indcrop.2019.111849.

[58] H. H. Fahmy , H. M. Raddy , M. H. A. El‐Sawah , and H. A. Gad , “Nanoemulsion of *Mentha Piperita* L. Essential Oil and Biotoxicity Against *Callosobruchus Maculatus* and *Callosobruchus Chinensis* ,” International Journal of Tropical Insect Science 46 (2026): 1255–1265, 10.1007/s42690-026-01760-3.

[59] H. A. Gad , H. H. Fahmy , H. M. Raddy , and M. H. A. El‐Sawah , “Essential Oil and Its Nanoemulsion of *Ocimum Basilicum*: Chemical Characterization and Insecticidal Activity Against Two Bruchid Beetles,” Journal of Stored Products Research 117 (2026): 103036, 10.1016/j.jspr.2026.103036.

[60] E. Mazzara , E. Spinozzi , F. Maggi , et al., “Hemp (*Cannabis sativa* cv. Kompolti) Essential Oil and Its Nanoemulsion: Prospects for Insecticide Development and Impact on Non‐Target Microcrustaceans,” Industrial Crops and Products 203 (2023): 117161, 10.1016/j.indcrop.2023.117161.

[61] F. Kalateh‐Seifari , H. Ahari , and S. Moradi , “A Review on the Food‐Based Applications of Nanometric Plant‐Based Essential Oils: Nanoencapsulation and Nanoemulsion Production Challenges,” Food and Bioprocess Technology 18 (2025): 6095–6115, 10.1007/s11947-025-03908-4.

[62] K. K. Nadarajah , “Nanotechnology in Crop Protection: Innovations for Sustainable Agriculture,” Agri‐Nanotechnology: Innovations for Sustainable Agriculture and Environmental Restoration (2025): 171–205, 10.1007/978-981-96-9756-4_7.

